# Fecal *Enterobacteriales* enrichment is associated with increased in vivo intestinal permeability in humans

**DOI:** 10.14814/phy2.13649

**Published:** 2018-04-02

**Authors:** Camilla Pedersen, Umer Z. Ijaz, Edith Gallagher, Felicity Horton, Richard J. Ellis, Etana Jaiyeola, Thibaut Duparc, David Russell‐Jones, Paul Hinton, Patrice D. Cani, Roberto M. La Ragione, M. Denise Robertson

**Affiliations:** ^1^ Faculty of Health and Medical Sciences University of Surrey Guildford United Kingdom; ^2^ School of Engineering University of Glasgow Glasgow United Kingdom; ^3^ Medical Physics ‐ Nuclear Medicine Royal Surrey County Hospital Guildford United Kingdom; ^4^ Animal and Plant Health Agency Addlestone United Kingdom; ^5^ WELBIO ‐ Walloon Excellence in Life Sciences and BIOtechnology Louvain Drug Research Institute Université Catholique de Louvain Brussels Belgium; ^6^ CEDAR Centre Royal Surrey County Hospital Guildford United Kingdom

**Keywords:** Endotoxemia, glucose control, intestinal microbiota, intestinal permeability, type 2 diabetes

## Abstract

Type 2 diabetes (T2D) has been linked with increased intestinal permeability, but the clinical significance of this phenomenon remains unknown. The objective of this study was to investigate the potential link between glucose control, intestinal permeability, diet and intestinal microbiota in patients with T2D. Thirty‐two males with well‐controlled T2D and 30 age‐matched male controls without diabetes were enrolled in a case–control study. Metabolic parameters, inflammatory markers, endotoxemia, and intestinal microbiota in individuals subdivided into high (HP) and normal (LP) colonic permeability groups, were the main outcomes. In T2D, the HP group had significantly higher fasting glucose (*P* = 0.034) and plasma nonesterified fatty acid levels (*P* = 0.049) compared with the LP group. Increased colonic permeability was also linked with altered abundances of selected microbial taxa. The microbiota of both T2D and control HP groups was enriched with *Enterobacteriales*. In conclusion, high intestinal permeability was associated with poorer fasting glucose control in T2D patients and changes in some microbial taxa in both T2D patients and nondiabetic controls. Therefore, enrichment in the gram‐negative order *Enterobacteriales* may characterize impaired colonic permeability prior to/independently from a disruption in glucose tolerance.

## Introduction

Impaired barrier function has been hypothesized to lead to the increased uptake of antigens of both dietary and bacterial origin from the intestinal lumen into the circulation, activating the innate immune system (Cani and Everard 2016). Animal studies suggest that gut microbiota composition and diet may be key factors. Increased fat content of the diet is associated with dysbiosis and increased circulating lipopolysaccharide (LPS), called metabolic endotoxemia and this has been associated with changes in the gut barrier function in mice (Cani et al. 2008, 2009; Kim et al. 2012). Increased circulating LPS and bacteria have been reported (Creely et al. 2007; Amar et al. 2011; Sato et al. 2014), in addition to well established dysbiosis in T2D patients (Larsen et al. 2010; Wu et al. 2010; Qin et al. 2012; Zhang et al. 2013; Remely et al. 2014, 2016; Lambeth et al. 2015; Lippert et al. 2017). The gram‐negative bacterial cell membrane component, LPS, is a potent ligand of the TLR‐4 receptor. TLR‐4 activation leads to increased levels of proinflammatory cytokines, such as TNF‐*α* and interleukins, and this systemic low‐grade inflammation is associated with insulin resistance (Cani et al. 2007). Administering LPS to animals and humans increases inflammatory markers and insulin resistance, corroborating a role for LPS in metabolic diseases characterized by low‐grade inflammation (Cani et al. 2007; Andreasen et al. 2010, 2011).

Impaired intestinal barrier function is suggested to be implicated in gastrointestinal and metabolic diseases, such as celiac disease, nonalcoholic fatty liver disease, and type 2 diabetes (T2D) (de Kort et al. 2011; Odenwald and Turner 2013; Cox et al. 2017). In a recent pilot study, T2D patients were found to have increased intestinal permeability (measured by urinary recovery of ^51^Cr‐EDTA) compared to healthy age‐, sex‐, and BMI‐matched subjects with small intestinal permeability correlating positively with circulating inflammatory markers (Horton et al. 2014). However, there were no associations between intestinal permeability and diet, anthropometrics or markers of metabolic control. Similarly, in a larger study a derived permeability risk score was higher in T2D patients compared to healthy controls (Cox et al. 2017). However, T2D patients and healthy individuals were not matched for BMI, age, or sex in this latter study (Cox et al. 2017).

However, it remains unclear what initiates this increased intestinal permeability. Moreover, to date, the link between intestinal permeability, oral antidiabetic drugs (e.g., metformin), biochemical indices, diet, and intestinal microbiota composition have not been thoroughly investigated.

In our previous work, we established that intestinal permeability in males with T2D was significantly higher than that of age‐, sex‐, and BMI‐matched healthy controls, however, the true clinical significance of this finding remains to be established in vivo (Horton et al. 2014). There is considerable variation in intestinal permeability with a proportion of T2D patients having intestinal permeability within what would be considered the healthy range, and similarly healthy individuals living with intestinal permeability values within the pathological range. For this reason, it is too simplistic to conclude that impaired permeability can be implicated in the etiology of T2D in all patients. In order to investigate this phenomenon, a group of T2D patients and age‐matched controls without diabetes were split into impaired intestinal permeability (high, HP) and normal permeability (low, LP) groups based on the upper 95% CI cut‐off of 1.58% for colonic permeability using the ^51^Cr‐EDTA intestinal permeability test data already established in healthy insulin‐sensitive individuals (Horton et al. 2014). The phenotypes of the colonic HP and LP groups were then compared in order to elucidate any potential link between intestinal permeability, gut bacterial profile, and systemic metabolism.

## Methods

This was a case–control study designed to investigate the role of intestinal bacteria and intestinal permeability in T2D patients and age‐matched control subjects without diabetes. The protocol was approved by the Central London NRES Committee (REC reference no. 11/LO/1141) and the University of Surrey Ethics Committee and was conducted according to the declaration of Helsinki.

### Subjects

Thirty‐two males with T2D and 30 males without diabetes (control subjects) were recruited through primary care and volunteer databases at the University of Surrey in 2012 and 2013. T2D had previously been diagnosed by the general practitioner of the participants. Women were excluded from this initial study due to the potential effect of the menstrual cycle on outcome measures in addition to the contraindication of administration of a radioactive tracer (^51^Cr‐EDTA) to healthy females who may be of child bearing age. All subjects provided written informed consent. Exclusion criteria included use of antibiotics in the previous 3 months, use of anti‐inflammatory medications (except a low‐dose aspirin (75 mg/day)), diuretics, inflammatory bowel disease, celiac disease, and irritable bowel disease. Mild dyslipidemia and hypertension were not considered reasons for exclusion. Renal function was tested (eGFR > 60) to ensure suitability for the ^51^Cr‐EDTA intestinal permeability test. The T2D patient group and control group were each split into HP and LP groups with a cut‐off value of 1.58% urinary ^51^Cr‐EDTA recovery (6–24 h after administration of the label).

### Dietary intakes

Subjects were instructed to avoid probiotic food items, fermented dairy such as yogurt and cheese in addition to prebiotic supplements for 2 weeks before the test day, this was assessed by a 7‐day diet diary. Amounts were estimated using normal household measurements. The diet diaries were analyzed in DietPlan6 (Forestfield Software Ltd, Horsham, UK).

### Fecal sample collection

Subjects collected fecal samples at home using universal sterile polystyrene containers. Fecal samples were initially stored at −20°C and subsequently at −80°C for long‐term storage.

### Blood pressure, anthropometrics, and biochemistry

After having emptied their bladder, body weight, and body composition was measured by bioimpedance (Tanita, Arlington Heights, IL, USA). Waist circumference was measured at the level of the naval with a tape measure. Blood pressure was measured after 5‐min rest and the mean of three readings calculated (Omron MX3 Plus, Omron Healthcare Europe, Milton Keynes, United Kingdom). A fasting blood sample (8 mL) was collected after a 10‐hour overnight fast at the CEDAR center of the Royal Surrey County Hospital. Blood was collected into EDTA tubes, serum tubes containing clotting activator and pyrogen‐free tubes for measurements of HbA1c, glucose, insulin, inflammatory markers, lipids, and LPS. Blood samples were centrifuged at 3000*g* at 4°C for 10 min and serum and plasma stored at −20°C or −80°C as appropriate.

### Intestinal permeability

Intestinal permeability was measured by 24 h urinary excretion of orally administered ^51^Cr‐EDTA as previously described (Horton et al. 2014). The ^51^Cr‐EDTA was administered after an overnight fast and patients were asked to collect all their urine in the next 24 h. The urine was collected into one container for the first 6 h and into a separate container for 6–24 h. The first 6 h of collection is considered to represent small intestinal permeability and the 6–24 h ^51^Cr‐EDTA recovery represents the colonic permeability. The adequacy of the urine collection was assessed using questionnaires on the completeness of the urine collection.

### Biochemical analyses

Whole blood glucose concentrations were measured immediately using the glucose oxidase method on the YSI 2300 STAT Plus (YSI Life Sciences, Fleet, UK) with a precision of 2% (or 0.2 mmol/L) and a linear range up to 50 mmol/L. The average intra‐assay CV was 4.8% and inter‐assay CV was 5.8%. Plasma insulin was analyzed in duplicate using a radioimmunoassay (Millipore, Billerica, MA) with an interassay CV of 12.6% and average intra‐assay CV of 7.7%. The sensitivity of the assay was 16.29 pmol/L. Serum hsCRP and HbA1c were measured by an accreditated laboratory, the Surrey Pathology Partnership and serum IL‐6 and TNF‐*α* were measured using a Luminex platform and Biorad bio‐plex kits and software. The limit of detection was 0.03 mg/L for hsCRP, 5 pg/mL for TNF‐*α* and 0.7 pg/ml for IL‐6. Triglycerides TAGs, total cholesterol, HDL cholesterol, and nonesterified fatty acids (NEFA) were measured on an ILab650 using commercially available kits (Randox Laboratories, UK, and Instrumentation Laboratory, UK). Average intra‐assay CVs were 1.4%, 1.9%, 0.6% and 1.0% and inter‐assay CVs were 1.9%, 3.0%, 1.1% and 1.8% and detection limits were 0.1 mmol/L, 0.22 mmol/L, 0.189 mmol/L, 0.072 mmol/L for TAGs, total cholesterol, HDL cholesterol, and NEFA, respectively. LDL cholesterol concentration was calculated using the Friedewald formula. LPS was measured in duplicate using Endosafe‐MCS (Charles River Laboratories, Lyon, France) as previously described (Everard et al. 2012). Samples were diluted from 1/20 to 1/200 and the samples were validated for recovery and coefficient of variation determination. The limit of detection was 0.005 EU/mL. Serum LPS binding protein (LBP) and sCD14 concentrations were measured using a solid‐phase enzyme‐linked immunosorbent assays (ELISA) according to the manufacturer's instructions (Hycult Biotechnology, Uden, the Netherlands). Sera were diluted 1/10 with the appropriate buffer and homogenized by vortex before further dilution to 1/200 (sCD14) and 1/1000 (LBP). Detection limits were 4.4 and 1.56 ng/mL and the average intra‐assay CVs were 3.9% and 8.5% and inter‐assay CVs were 19.6% and 15.5% for LBP and sCD14, respectively.

### DNA extraction

DNA was extracted from defrosted fecal samples using the PowerFecal™ DNA Isolation Kit (MO BIO Laboratories Inc., Carlsbad, CA, USA) according to the manufacturer's instructions. The DNA concentration and quality were measured by NanoDrop 2000 (Thermo Scientific) and Qubit 2.0 fluorometer (Invitrogen). Samples with a DNA concentration >50 ng/*μ*L was used for sequencing. Due to unsuccessful DNA extraction for some samples, bacterial data are only available for a subset of T2D patients (*n* = 23) and controls (*n* = 27).

### Amplification and high‐throughput sequencing

DNA amplification and sequencing were performed as previously described (Ellis et al. 2013). Briefly, the V4 and V5 region of the bacterial 16S rRNA gene was amplified from extracted DNA with universal primers (U515F: 5′‐GTGYCAGCMGCCGCGGTA and U927R: 5′‐CCCGYCAATTCMTTTRAGT). Forward fusion primers consisted of the GS FLX Titanium primer A and the library key (5′ ‐CATCTCATCCCTGCGTGTCTCCGACTCAG) together with one of a suite of sixteen 10‐base multiplex identifiers (MIDs 1–16) (Roche Diagnostics Ltd, UK). Reverse fusion primers included the GS FLX Titanium primer B and the library key (5′‐CCTATCCCCTGTGTGCCTTGGCAGTCTCAG). Amplification was performed with FastStart HiFi Polymerase (Roche Diagnostics Ltd, UK) using the following cycling conditions: 94°C for 3 min; 30 cycles of 94°C for 30 sec, 55°C for 45 sec, 72°C for 1 min; followed by 72°C for 8 min. Ampure XP magnetic beads (Beckman Coulter) were used for purification of amplicons. Amplicon concentration was assessed using the fluorescence‐based Picogreen assay (Invitrogen) and concentrations normalized before pooling. Amplicon pools were immobilized and amplified on beads by emulsion PCR using Lib‐L emPCR kits (Roche Diagnostics Ltd, UK). Unidirectional sequencing from the forward primer was performed on the 454 GS FLX Titanium platform according to the manufacturer's instructions (Roche Diagnostics Ltd, UK).

### Quantification of bacterial groups by quantitative PCR

Total bacteria and the bacterial groups *Bifidobacterium, Roseburia*,* Lactobacillus*,* Enterobacteriaceae*,* Clostridium leptum*, and *Clostridium coccoides* groups were quantified by quantitative PCR (qPCR) on a QuantStudio 7 Flex Real‐time system (Life technologies, USA). The qPCR primers are described in Table 1. A typical 20 *μ*L qPCR reaction contained 0.3 *μ*mol/L of each (forward and reverse) primer, 10 *μ*L GoTaq qPCR master mix, 7.8 *μ*L of nuclease‐free water and 5–20 ng of template genomic DNA extract. The qPCR cycling protocol consisted of 1× initial denaturation cycle at 95°C for 2 min followed by 40× denaturation at 95°C for 15 sec, annealing at 55°C for 30 sec, and extension at 72°C for 30 sec; fluorescence was measured after each cycle and 1× dissociation −60–95°C.

**Table 1:** Primers used for qPCR

Target	Primer name	Sequence	Product bp
*Clostridium leptum* subgroup	C‐leptF	GCACAAGCAGTGGAGT	239
	C‐leptR	CTTCCTCCGTTTTGTCA	
*Clostridium coccoides* subgroup	C‐cocF	AAATGACGGTACCTGACTAA	440
	C‐cocR	CTTTGAGTTTCATTCTTGCGAA	
*Roseburia*	RosF	TACTGCATTGGAAACTGTCG	230
	RosR	CGGCACCGAAGAGCAAT	
*Lactobacillus* group	LacF	AGCAGTAGGGAATCTTCCA	341
	LacR	CACCGCTACACATGGAG	
*Bifidobacterium*	BifF	GCGTGCTTAACACATGCAAGTC	126
	BifR	CACCCGTTTCCAGGAGCTATT	
All bacteria	UnivF	TCCTACGGGAGGCAGCAGT	466
	UnivR	GACTACCAGGGTATCTAATCCTGTT	
*Enterobacteriaceae*	EcoF	CATTGACGTTACCCGCAGAAGAAGC	190
	EcoR	CTCTACGAGACTCAAGCTTGC	

Quantitation of each target in the samples was determined based on a standard curve of each target using purified target DNA template. A 10‐fold dilution series ranging from 1 × 10^4^–1 × 10^8^ copies of each target gene was prepared in nuclease‐free water and analyzed in triplicate. The test samples were analyzed in 96‐well plates (MicroAMP Optical plates, Life Technologies, USA), along with the standard. The qPCR software generated the standard curve (based on the average of each standard) and computed the template concentrations. The amplification of a single product by the primer sets used was confirmed by analysis of the dissociation profile of each target and agarose gel electrophoresis of a standard PCR reaction using each primer set, the same cycling conditions and DNA template.

### Bioinformatics and statistical analysis

The sequences were processed in Qiime (Caporaso et al. 2010) using the AmpliconNoise (Quince et al. 2011) pipeline that utilizes flowgram information of the sequences to correct for errors. The samples were demultiplexed by exact matching of both barcode and primer and the sequences filtered and trimmed based on identification of low‐quality signals (Quince et al. 2009). The filtered flowgrams were clustered to remove platform‐specific errors and converted into sequences using the PyroNoise algorithm. The sequences had barcodes and degenerate primers removed prior to trimming at 500 base pairs (bp). They were then further clustered by SeqNoise to remove PCR single base errors. In the final step, the Perseus algorithm was used to identify chimeras. The denoised sequences were classified using the standalone RDP classifier (Wang et al. 2007). From this, taxa frequencies at five different levels: Phylum, Class, Order, Family and Genus; were calculated. Additionally, a nonsupervised approach was used, operational taxonomic units (OTUs) were generated at 3% divergence following pair‐wise global sequence alignment and hierarchical clustering with an average linkage algorithm. To improve resolution at the OTU level, sequences were also compared with databases at the NCBI website (http://blast.ncbi.nlm.nih.gov/Blast.cgi). Further statistical analyses were performed in R using the tables and data generated as above as well as the meta‐data associated with the study. For community analyses (including alpha and beta diversity analyses and permutation ANOVA using distance measures (adonis function)) we used the vegan (http://cran.r-project.org/web/packages/vegan/) package. To calculate Unifrac distances (that account for phylogenetic closeness), we used the phyloseq (McMurdie and Holmes 2013), ape (Paradis et al. 2004), and phangorn (Schliep 2011) packages. To determine significant differences in bacterial abundances between the groups, we used DESeqDataSetFromMatrix() function from DESeq package with a significance value cut‐off of 0.05. This function allows negative binomial GLM fitting (as abundance data from metagenomic sequencing is overdispersed) and Wald statistics for abundance data and identifies species with log‐fold changes between different conditions. General scripts and tutorials for the above analyses are available at http://userweb.eng.gla.ac.uk/umer.ijaz#bioinformatics.

Data are presented as mean and SD or median and interquartile range as appropriate. T2D, overweight/obese (OW), and normal weight (NW) groups were compared using a one‐way ANOVA with Sidak corrections for multiple comparisons using OW controls as the control group. Nonparametric data were tested using the Kruskal–Wallis test with Dunn's post hoc tests. Pair‐wise comparisons of HP and LP groups were performed using unpaired *t* tests for parametric data and Mann–Whitney tests for nonparametric data. RT qPCR total bacteria values are presented as log_10_ of values. Values for bacterial groups were normalized to total bacteria. Where appropriate, p‐values were adjusted for multiple corrections using the Benjamini–Hochberg method (Benjamini and Hochberg 1995). Data were analyzed in GraphPad Prism 6, SPSS 22 and R. The level of significance was set at *P* < 0.05.

## Results

In an exploratory analysis the 30 nondiabetic controls were split into two groups: NW controls (*n* = 14) matched on age and OW controls (*n* = 16) matched to the diabetes patients on age and BMI. The anthropometric, dietary, and clinical data for T2D, OW controls, and NW controls are shown in Table 2.

**Table 2:** Anthropometric characteristics, clinical parameters, and dietary intakes in T2D patients, overweight (OW), and normal weight (NW) controls. Data are presented as means (SD) or medians (interquartile range)

	T2D	Control OW	Control NW	*P*‐value
*n*	32	16	14	–
Age (years)	57.9 (6.2)	57.3 (7.2)	56.6 (7.4)	ns
Body weight (kg)	88.4 (12.8)	91.2 (8.0)	68.3 (6.1)^a^	<0.0001
BMI (kg/m^2^)	28.4 (25.8–30.8)	28.3 (26.7–29.8)	22.5 (20.4–23.6)^a^	<0.0001
Body fat (%)	25.9 (4.9)	26.2 (4.6)	17.6 (5.0)^a^	<0.0001
Waist circumference (cm)^b^	102.1 (10.4)	103.3 (9.4)	84.7 (6.3)^a^	<0.0001
Fasting glucose (mmol/L)	6.0 (5.3–7.2)^a^	4.7 (4.4–5.0)	4.4 (4.2–4.9)	<0.0001
Fasting insulin (pmol/L)	80.1 (49.3–110.0)	59.9 (47.1–107.8)	41.6 (35.0–52.0)^a^	0.0032
HOMA2%B	102.0 (43.6)^a^	151.5 (59.3)	109.8 (22.3)^a^	0.0023
HOMA2%S	55.8 (41.8–84.4)	81.6 (42.7–102.7)	116.3 (91.5–141.8)^a^	0.0012
HOMA2 IR	1.8 (1.2–2.4)	1.2 (1.0–2.4)	0.9 (0.7–1.1)^a^	0.0011
HbA1c (mmol/mol)	49.0 (9.5)	n/a	n/a	–
HbA1c (%)	6.6 (0.9)	n/a	n/a	–
BP sys (mm Hg)^b^	135 (11)	133 (12)	118 (8)^a^	<0.0001
BP dia (mm Hg)^b^	84 (7)	86 (7)	78 (8)^a^	0.0052
Total Cholesterol (mmol/L)	3.4 (0.9)^a^	5.1 (1.0)	5.1 (0.9)	<0.0001
HDL Cholesterol (mmol/L)	1.0 (0.3)	1.1 (0.3)	1.4 (0.4)^a^	0.0009
LDL Cholesterol (mmol/L)	2.0 (0.8)^a^	3.5 (0.8)	3.3 (0.7)	<0.0001
NEFA (mmol/L)	0.67 (0.25)^a^	0.45 (0.21)	0.47 (0.25)	0.0047
TAGs (mmol/L)	1.00 (0.40)	1.03 (0.45)	0.93 (0.22)	ns
^51^Cr‐EDTA 0–6 h (%)	1.6 (0.9)	1.4 (0.7)	1.5 (0.6)	ns
^51^Cr‐EDTA 6–24 h (%)	1.4 (1.1–2.1)	1.3 (0.9–1.6)	1.6 (1.3–1.9)	ns
^51^Cr‐EDTA total (%)	3.4 (1.6)	2.7 (1.0)	3.0 (0.9)	ns
LPS (EU/mL)^b^	0.63 (0.48–1.15)	0.49 (0.37–0.85)	0.84 (0.73–1.21)^a^	ns
LBP (*μ*g/mL)	10.8 (8.7–13.0)	12.1 (10.7–13.4)	9.0 (7.4–10.2)^a^	0.0112
sCD14 (*μ*g/mL)	1.0 (0.8–1.2)	1.0 (0.8–1.3)	0.9 (0.8–1.1)	ns
hsCRP (mg/L)	1.36 (0.55–2.06)	1.32 (0.81–3.35)	0.44 (0.26–1.23)^a^	0.0194
IL‐6 (pg/mL)	11.6 (7.0–13.9)	19.6 (7.4–36.0)	7.0 (5.5–18.4)	ns
TNF‐*α* (pg/mL)	13.0 (5.0–22.3)	7.0 (5.0–28.9)	6.0 (5.0–7.3)	ns
Shannon index^c^	3.294 (2.981–3.580)	3.186 (3.139–3.387)	3.348 (3.037–3.695)	ns
Observed Richness^c^	76.71 (66.55–85.07)	78.80 (63.91–96.15)	85.57 (68.58–104.80)	ns
Pielou's evenness^c^	0.706 (0.657–0.751)	0.677 (0.681–0.707)	0.696 (0.635–0.749)	ns
Energy intake (kJ/day)	8672 (2054)	9939 (1710)	9662 (2323)	ns
Total carbohydrate (% of energy)	41.4 (7.6)	39.8 (5.2)	45.3 (6.8)	ns
Sugars (% of energy)	14.6 (5.3)	17.2 (4.5)	19.9 (5.4)	0.0086
Protein (% of energy)	16.4 (3.1)	15.1 (2.0)	14.8 (1.6)	ns
Total fat (% of energy)	36.7 (5.6)	39.9 (5.5)	34.6 (5.8)^a^	0.0418
Saturated fat (% of energy)	12.3 (2.5)^a^	14.6 (3.1)	12.9 (3.1)	0.0279
Alcohol (g/day)	9.3 (0.4–25.5)	13.1 (5.9–22.6)	12.2 (9.4–19.1)	ns
Dietary fiber (g/day)	22 (5)	23 (7)	27 (12)	ns
Sodium (mg/day)	3138 (834)	3398 (777)	2833 (713)	ns

n/a, not measured. NW group, *n* = 13 for dietary data. BP, blood pressure (sys: systolic, dia: diastolic).

a Significantly different from OW Control group (*P* < 0.05).

b *n* = 31 for the T2D group.

c *n* = 20, *n* = 13 and *n* = 12 for the T2D, OW and NW group, respectively.

Six of the T2D patients were diet/exercise controlled; the remaining 26 patients were on various oral antidiabetic medications (n): Metformin (12), sulfonylureas (1), DPP4 inhibitor (1), metformin and DPP4 inhibitor (4), metformin and sulfonylureas (5), sulfonylureas and DPP4 inhibitor (1), metformin, sulfonylureas, and DPP4 inhibitor (1), and metformin, DPP4 inhibitor and thiazolidinedione (1). All patients had been on a stable treatment for at least 3 months prior to taking part in the study. Other medications taken by T2D patients included low lipid lowering medications (mainly statins) (21), blood pressure lowering medications (16), omeprazole (3), levothyroxine (2), fenofibrates (2), Betahistine hydrochloride (1) and medications for incontinence (2), benign prostate hyperplasia (3), fungal infection (2), hay fever (2), asthma (inhaler) (1), and depression (1).

One NW and one OW control subject were taking statins and two OW controls were taking blood pressure medication. Other medications used in the control groups were asthma inhalers (4), benign prostate hyperplasia medication (1), antidepressant (1), mysoline (1), epilim (1), becotide (1), beconase (1), qvar (1), and dutasteride (1).

### Biochemical outcomes

Fasting glucose was significantly higher in the T2D patients compared with OW controls. HOMA %B was significantly higher in OW controls compared with both T2D patients and NW controls (*P* = 0.0013 and *P* = 0.0268, respectively), whereas fasting insulin concentrations, insulin sensitivity (HOMA %S), and insulin resistance (HOMA IR) were only significantly different between control groups (*P* = 0.0418, *P* = 0.0385, and *P* = 0.0383, respectively).

Serum total and LDL cholesterol concentrations were lower and NEFA concentrations higher in T2D patients compared with OW controls (*P* < 0.0001, *P* < 0.0001, and *P* = 0.0092, respectively). HDL cholesterol was significantly lower in OW controls than in NW controls (*P* = 0.0325). LBP (*P* = 0.0055) and hsCRP concentrations (*P* = 0.0163) were higher, but LPS concentrations were lower (*P* = 0.0432) in OW controls compared with NW controls. There were no significant differences between groups in TGs, sCD14, IL‐6, and TNF‐*α* concentrations, although IL‐6 concentrations tended to be higher in the OW controls compared to NW controls (*P* = 0.0669) (Table 2).

### Intestinal permeability

There were no significant differences in the recovery of ^51^Cr‐EDTA in urine between groups (Table 2).

### Dietary assessment

No significant differences in energy intake between groups were observed (Table 2). However, T2D patients reported significantly lower sugar intake (g/day), total fat (g/day) and saturated fat (g/day and percentage of energy) intakes than OW controls (*P* = 0.0252, *P* = 0.0139, *P* = 0.0013, and *P* = 0.0156, respectively). Fat percentage of energy was higher in the OW control group than in the NW control group (*P* = 0.0286).

### Bacterial community structure

Due to unsuccessful DNA extraction (DNA concentration <50 ng/mL) for a number of samples, the qPCR dataset consisted of *n* = 23 for the T2D group, *n* = 15 for the OW group and *n* = 12 for the NW group. After excluding samples with <500 bp, the next‐generation sequencing dataset consisted of 20, 13, and 12 for the T2D, OW, and NW group, respectively.

**Figure 1:**
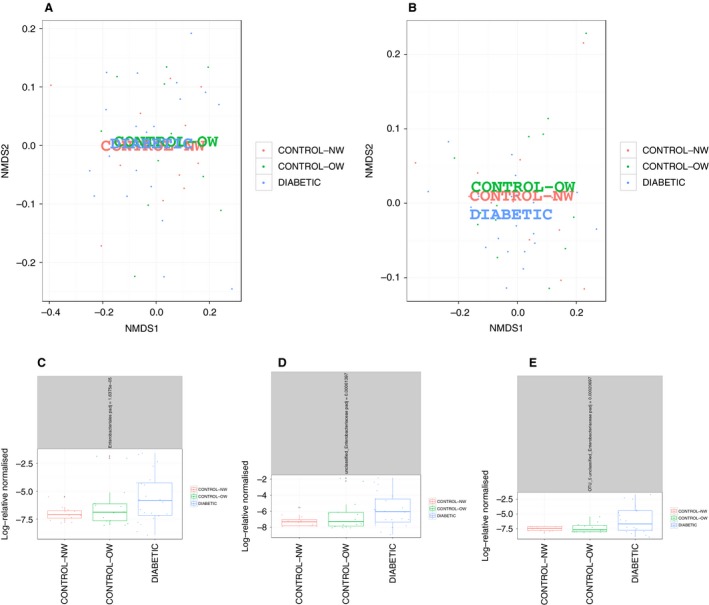
Unweighted (A) and weighted (B) UniFrac distances in T2D patients and normal weight (NW) and overweight/obese (OW) controls. There were significant differences between groups in the order *Enterobacteriales*, genus unclassified *Enterobacteriaceae* and *Escherichia coli* (OTU 5) between normal weight (NW) controls (*n* = 12), overweight/obese (OW) controls (*n* = 13), and T2D patients (*n* = 20) (C–E). FDR adjusted *P*‐values are displayed in the header of the panels.

Consistent with previous reports the predominating phyla were Bacteroidetes and Firmicutes followed by unclassified bacteria and Proteobacteria. There were no significant differences in alpha‐diversity, richness, or evenness indices between groups (Table 2). Ordination plots did not show any clustering of T2D patients and control groups (Fig. 1A and B). However, comparison of bacterial abundances showed significant differences at the order, genus and OTU levels with higher abundance of *Enterobacteriales*, unclassified *Enterobacteriaceae*, and *Escherichia coli* (OTU 5), respectively, in T2D patients (Fig. 1C–E). No significant differences between groups were found at the phylum, class, or family levels in the next‐generation sequencing dataset.

**Figure 2:**
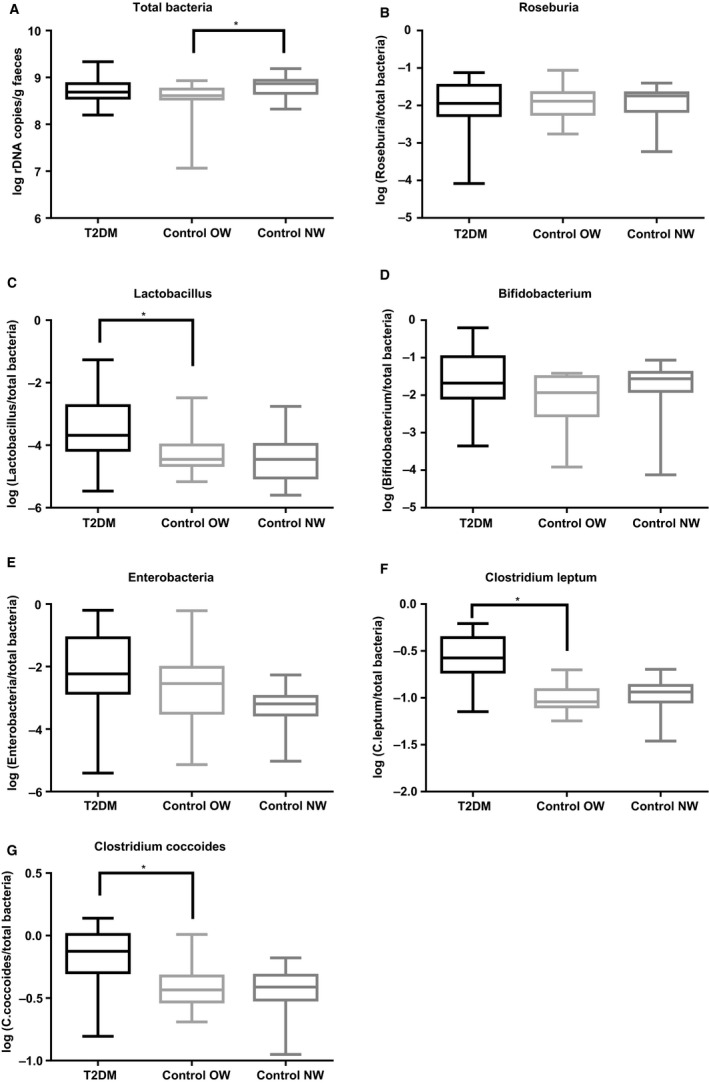
Total bacteria and selected bacterial groups were quantified by real‐time qPCR (median, interquartile ranges, and min and max values, *n* = 23, *n* = 15 and *n* = 12 for T2D, OW, and NW control groups, respectively). Although post hoc tests showed that total bacteria were significantly higher in control NW compared to control OW (*P* = 0.030), there was only a trend toward a main effect (*P* = 0.0516) (A). Abundances of *Lactobacillus* spp. and *Clostridium leptum* and *Clostridium coccoides* clusters were significantly different between groups (*P* = 0.0062, *P* < 0.0001 and *P* < 0.0001, respectively) with significantly higher levels in T2D compared to control OW (*P* = 0.0183, *P* < 0.0001 and *P* = 0.004, respectively) (C, F and G). While *Roseburia* spp. and *Bifidobacterium* spp. did not differ between groups (*P* = 0.9835 and *P* = 0.1660) (B and D), there was a trend toward a differences in *Enterobacteriaceae* between groups (*P* = 0.0504) (E).

Real‐time PCR measurements showed that *Lactobacillus* spp., *C. leptum,* and *C. coccoides* levels were significantly higher in T2D patients compared with OW controls (Fig. 2). Total bacteria was significantly higher in NW controls than in OW controls, but no other significant differences between OW and NW control groups were found. However, there was a trend for a difference in *Enterobacteriaceae* levels with lowest levels observed in NW controls and highest levels in the T2D group.

### Correlations between clinical outcomes

**Figure 3:**
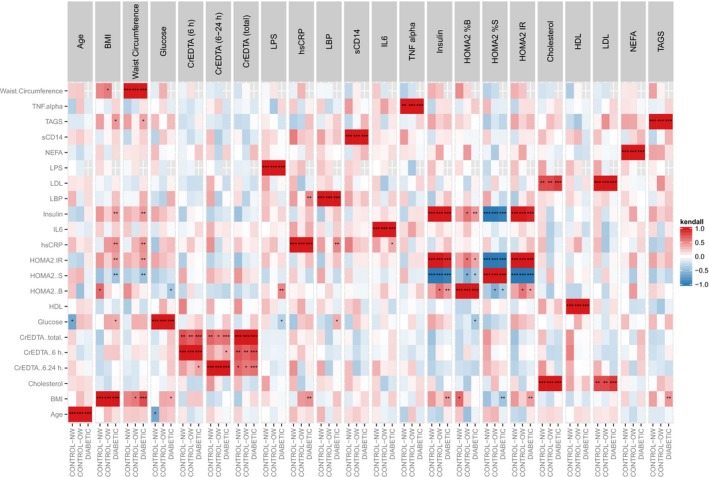
Correlation heat maps showing associations (Kendall's rank correlations) between clinical outcomes (adjusted for multiple testing). Due to missing data for some participants, correlations coefficients were not calculated for waist circumference and LPS. Normal weight (NW) controls: *n* = 12, overweight/obese (OW) controls: *n* = 13, and T2D patients: *n* = 20.

Intestinal permeability did not correlation with clinical outcomes in any group (Fig. 3). However, in T2D patients LPS correlated with beta‐cell function (*r* = 0.52, adj. *P* = 0.0025) and fasting glucose (*r* = −0.36, adj. *P* = 0.049), whereas hsCRP correlated with LBP (*r* = 0.52, adj. *P* = 0.0019), IL‐6 (*r* = 0.36, adj. *P* = 0.0416), and BMI (*r* = 0.48, adj. *P* = 0.0034) and LBP correlated with fasting glucose (*r* = 0.35, adj. *P* = 0.0488). Furthermore, waist circumference and BMI correlated positively with insulin resistance, TGs, and fasting insulin and inversely with insulin sensitivity. Few significant correlations were found in the control groups.

### Comparison of high permeability and low (normal) permeability groups

**Figure 4:**
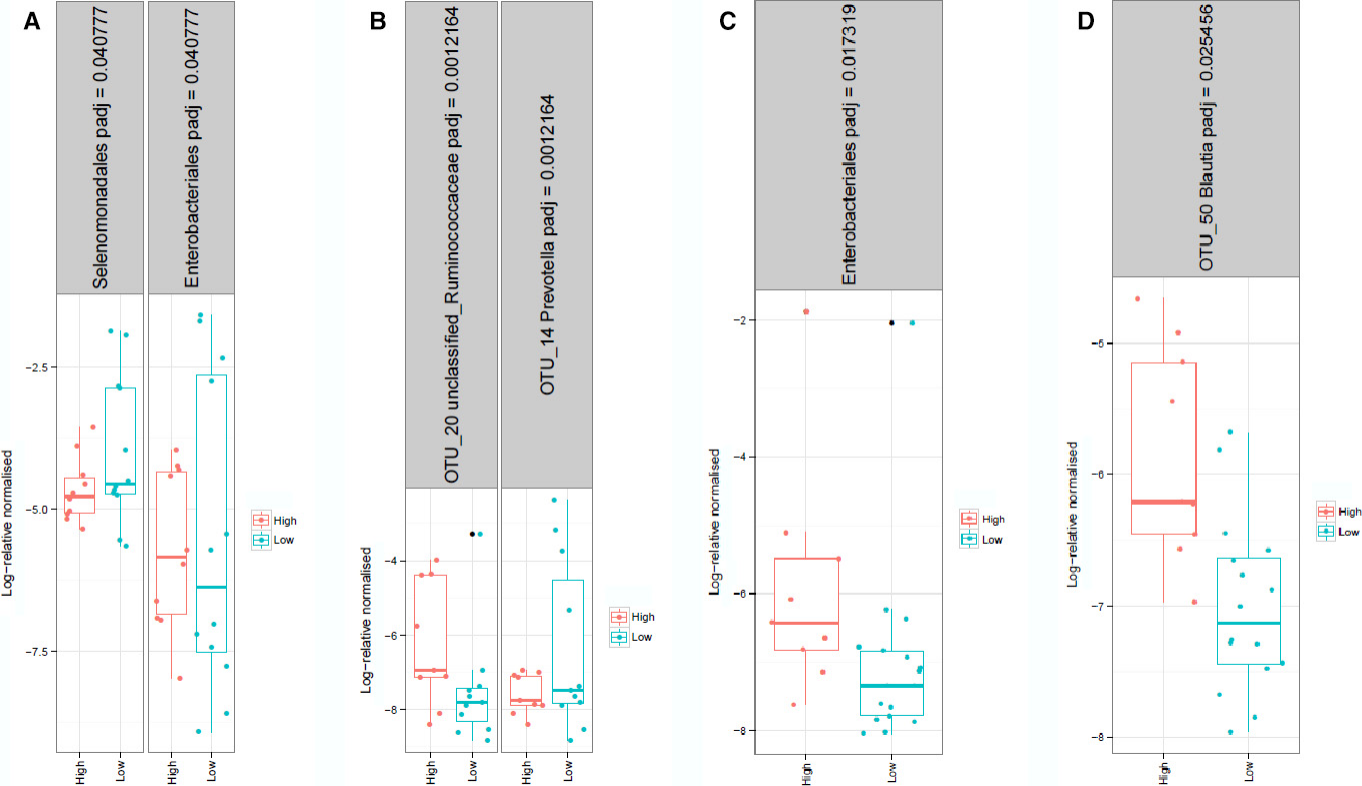
Differences in abundances of several bacteria in type 2 diabetes patients (A and B) and controls (C and D) colonic HP and LP groups were found. Taxonomic annotation and adjusted *P*‐values are displayed in the headers. T2D HP:* n* = 9, T2D LP:* n* = 11, Control HP:* n* = 9, Control LP:* n* = 16.

As the density of bacteria is highest in the colon we explored whether clinical parameters, dietary intakes (Tables 3 and 4) and intestinal bacterial abundances (Fig. 4) differed between those with high and normal colonic permeability. Time since diagnosis of diabetes was similar in the LP and HP diabetes groups (mean: 4.6 years (range: 0.1–10 years) and 4.7 years (range: 0.5–11 years), respectively).

**Table 3:** Clinical outcomes in normal (LP) and high (HP) colonic permeability groups. Data are presented as means (SD) or median (interquartile range)

	T2D patients			Controls		
	LP	HP	*P*‐value	LP	HP	*P‐*value
*n*	19	13	–	19	11	–
^51^Cr‐EDTA 6–24 h (%)	1.17 (0.99–1.36)	2.19 (1.81–3.10)	<0.0001	1.16 (0.90–1.47)	1.87 (1.64–2.09)	<0.0001
Age (years)	58.2 (6.3)	57.4 (6.2)	ns	56.6 (7.0)	57.5 (7.9)	ns
Weight (kg)	86.6 (12.7)	91.0 (12.9)	ns	84.6 (13.6)	73.5 (10.7)	0.029
BMI (kg/m^2^)	27.5 (3.3)	29.7 (3.9)	ns	26.6 (4.5)	24.2 (3.0)	ns
Body fat (%)	25.0 (4.7)	27.4 (5.1)	ns	22.7 (7.3)^a^	20.9 (4.7)	ns
Waist circumference (cm)	100.1 (10.1)^a^	104.9 (10.6)	ns	97.6 (14.0)	89.5 (6.9)	ns
Blood pressure, systolic (mm Hg)	135 (10)^a^	135 (12)	ns	127 (10)	125 (16)	ns
Blood pressure, diastolic (mm Hg)	85 (6)^a^	84 (8)	ns	82 (9)	82 (9)	ns
HbA1c (mmol/mol)	47.4 (6.6)	51.2 (12.4)	ns	n/a	n/a	–
HbA1c (%)	6.5 (0.4)	6.8 (1.3)	ns	n/a	n/a	–
Fasting glucose (mmol/L)	5.8 (1.1)	6.7 (1.1)	0.034	4.7 (0.6)	4.6 (0.6)	ns
Fasting insulin (pmol/L)	81.4 (47.9–111.7)	70.0 (50.3–132.2)	ns	51.5 (41.9–77.7)	42.3 (35.6–53.4)	ns
HOMA %B	112.5 (44.5)	86.7 (39.0)	ns	127 (109–147)	111 (86–134)	ns
HOMA %S	54.1 (41.8–87.2)	59.9 (35.2–85.1)	ns	92.6 (61.1–111.5)	109.3 (88.3–140.6)	ns
HOMA IR	1.88 (1.15–2.40)	1.67 (1.19–2.97)	ns	1.08 (0.90–1.64)	0.91 (0.71–1.13)	ns
Total cholesterol (mmol/L)	3.5 (1.0)	3.3 (0.7)	ns	5.0 (1.0)	5.4 (0.7)	ns
LDL cholesterol (mmol/L)	2.0 (0.9)	1.9 (0.6)	ns	3.3 (0.8)	3.5 (0.7)	ns
HDL cholesterol (mmol/L)	1.0 (0.2)	1.0 (0.3)	ns	1.2 (0.4)	1.5 (0.4)	0.040
NEFA (mmol/L)	0.60 (0.22)	0.77 (0.26)	0.049	0.47 (0.20)	0.45 (0.28)	ns
TAGs (mmol/L)	1.06 (0.44)	0.91 (0.32)	ns	0.95 (0.79–1.13)	0.86 (0.73–1.03)	ns
hsCRP (mg/L)	1.3 (0.6–1.7)	1.5 (0.5–4.4)	ns	2.7 (6.0)	1.5 (1.5)	ns
IL‐6 (pg/mL)	8.5 (7.0–12.6)	13.3 (5.5–14.9)	ns	7.0 (5.5–20.3)	19.6 (8.5–30.3)	ns
TNF‐*α* (pg/mL)	13.0 (5.0–22.3)	8.2 (5.0–17.7)	ns	7.0 (5.0–13.0)	7.0 (5.0–13.0)	ns
LPS (EU/mL)	0.93 (0.50–1.38)	0.49 (0.38–1.05)^b^	ns	0.68 (0.40–1.08)	0.84 (0.48–1.00)	ns
LBP (*μ*g/mL)	11.0 (3.1)	10.3 (4.7)	ns	10.5 (7.6–13.3)	10.3 (9.0–11.5)	ns
sCD14 (*μ*g/mL)	1.03 (0.78–1.37)	0.94 (0.77–1.09)	ns	0.88 (0.82–1.11)	1.11 (0.82–1.29)	ns
Metformin (*n*/%)	14/73.7	9/69.2	–	n/a	n/a	–
Statins (*n*/%)	14/73.7	7/53.8	–	1/5.3	1/9.1	–
BP medication (*n*/%)	10/52.3	8/61.5	–	2/10.5	0/0	–
Aspirin (*n*/%)	3/15.8	3/23.1	–	0/0	0/0	–

a *n* = 18.

b *n* = 12.

**Table 4:** Dietary intakes based on 7‐day diet diaries (means (SEM) or median (interquartile range))

	T2D patients			Controls		
	LP	HP	*P*‐value	LP	HP	*P‐*value
*n*	19	13		18	11	
Energy (kJ)	9187 (1849)	7921 (2176)	ns	10476 (1818)	8733 (1796)	0.018
Dietary fiber (g/day)	23 (6)	20 (5)	ns	25 (22–34)	17 (16–24)	0.022
Alcohol (g/day)	13 (13)	12 (12)	ns	15 (9)	13 (9)	ns
Sodium (mg/day)	3249 (947)	2975 (635)	ns	3322 (3086–3659)	2530 (1903–3580)	ns
Total carbohydrate (% of energy)	41.7 (9.0)	40.8 (5.1)	ns	43.0 (7.0)	41.1 (5.6)	ns
Sugars (% of energy)	15.4 (6.1)	13.5 (3.7)	ns	19.3 (5.4)	17.0 (4.2)	ns
Protein (% of energy)	15.8 (3.3)	17.2 (2.5)	ns	15.2 (2.2)	14.6 (1.1)	ns
Total fat (% of energy)	36.5 (6.0)	37.0 (5.3)	ns	36.8 (6.3)	38.8 (5.9)	ns
Saturated fat (% of energy)	12.3 (2.2)	12.1 (2.9)	ns	13.3 (2.5)	14.7 (4.1)	ns
Alcohol (% of energy)	3.8 (0–8.7)	3.1 (0.3–6.5)	ns	4.2 (2.8)	4.3 (3.1)	ns

### Anthropometrics, biochemistry, and diet

T2D patients in the HP group had significantly higher fasting glucose and NEFA concentrations than those in the LP group. No significant differences in inflammatory markers or dietary intakes were detected between groups, although there were a trend toward a lower total carbohydrate intake and higher BMI in the HP group (Table 4). In contrast, in the controls, the HP group had significantly lower body weight, higher HDL cholesterol concentration and lower total energy and absolute sugar, protein and dietary fiber intakes than the LP group. Inflammatory markers and other glucose tolerance markers did not differ between control LP and HP groups (Table 3).

### Differences in intestinal bacterial abundances

No significant differences in overall bacterial community composition between LP and HP for both T2D and control groups were found (adonis analysis, *P* > 0.7). As for bacteria abundances, the order *Selenomonadales* and *Prevotella* (OTU 14) were higher and *Enterobacteriales* and an unclassified *Ruminococcaceae* (OTU 20) were lower in the LP group compared to the HP T2D group. In controls, *Enterobacteriales* and *Blautia* (OTU 50) were enriched in the HP group. Accessions numbers for OTUs are provided in Table 5.

**Table 5:** RDP and NCBI taxonomy and annotation. When more than one match (99–100%) was found, three accession numbers are listed

OTU	RDP taxonomy	NCBI taxonomy	Query cover/identity	NCBI accession no.
OTU_3	Unclassified *Bacteriodes*	*Bacteroides vulgatus* ATCC 8482	100/99	NC_009614
OTU_5	Unclassified *Enterobacteriaceae*	*Escherichia coli* O83:H1 str. NRG 857C, *Escherichia coli* str. K‐12 substr. MG1655, *Escherichia coli* UMN026 chromosome	100/99	NC_017634 NC_000913.3 NC_011751
OTU_7	Unclassified Bacteroidetes	No 99–100% match.		
OTU_14	*Prevotella*	No 99–100% match.		
OTU_20	Unclassified *Ruminococceae*	No 99–100% match.		
OTU_44	*Clostridium* XIVa	No 99–100% match.		
OTU_50	*Blautia*	No 99–100% match.		
OTU_72	Unclassified_*Ruminococcaceae*	No 99–100% match.		
OTU_93	Unclassified_*Lachnospiraceae*	No 99–100% match.		
OTU_109	*Faecalibacterium*	No 99–100% match.		
OTU_129	*Parasuttarella*	*Parasutterella excrementihominis* YIT 11859 genomic scaffold Scfld40	100/100	NZ_GL883702
OTU_131	Unclassified *Lachnospiraceae*	No 99–100% match.		
OTU_151	Unclassified *Lachnospiraceae*	No 99–100% match.		
OTU_177	*Sutterella*	*Sutterella wadsworthensis* 2_1_59BFAA	100/99	NZ_JH815522, NZ_JH815517, NZ_JH815517
OTU_180	*Ruminococcus*	*Ruminococcus callidus* ATCC 27760	100/100	NZ_KI260393
OTU_195	*Clostridium* XI	*Clostridium* sp. 01 genomic scaffold, scaffold00220	99/99	NZ_HG529443
OTU_329	Unclassified Firmicutes	No 99–100% match.		

*Enterobacteriales* enrichment therefore appears to characterize high colonic permeability in both T2D patients and healthy controls (Fig. 4). No significant differences at the phylum level, in diversity, richness, evenness indices (Table 6) or any of the bacterial groups measured by qPCR between HP and LP groups were found (data not shown).

### Retrospective analysis: effect of metformin on gut microbiota in diabetes patients

**Figure 5:**
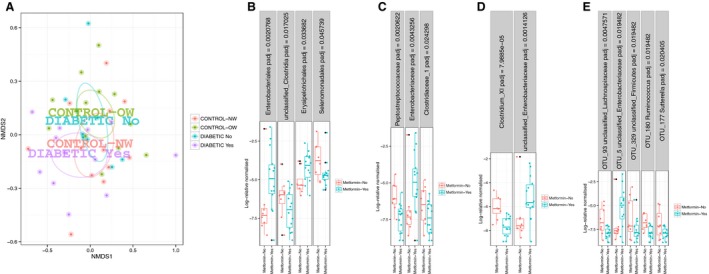
(A) Bray–Curtis distances in control groups (OW: overweight/obese (*n* = 13), NW: normal weight (*n* = 12) and metformin‐treated type 2 diabetes patients (Diabetic – Yes, *n* = 13) and diabetes patients not treated with metformin (Diabetic – No, *n* = 7)). The circles show the 95% confidence intervals and the subgroup legends in the plot represent the mean value of each group. Significant differences in bacterial abundances between groups were found at the order (B), family (C), genus (D), and (E) OTU levels.

While this study was underway, several studies were published indicating that metformin has a profound effect on the gut microbiota composition in both animal and human studies (Karlsson et al. 2013; Napolitano et al. 2014; Shin et al. 2014; Forslund et al. 2015; de la Cuesta‐Zuluaga et al. 2017). Although the study was not powered to look at the effects of medications, an exploratory investigation into the effects of metformin was undertaken. Bacterial data were available for seven nonmetformin‐treated patients and 13 metformin‐treated patients. We found no significant effect of metformin on overall microbiota composition (*P* > 0.5, Fig. 5A). However, the orders *Enterobacteriales* and *Erysipelorichales*, the family *Enterobacteriaceae* and an unclassified Enterobactericeae at the genus level and *E*. *coli* (OTU 5) at the species‐like level were significantly enriched following metformin treatment (Fig. 5B–E). In contrast, the orders *Selenomonadales* and unclassified *Clostridia*, the families *Peptostreptococcaceae* and *Clostridiaceae* 1, the genus *Clostridium* cluster XI and three OTUs belonging to the Firmicutes in addition to *Sutterella* (Proteobacteria) were decreased in metformin‐treated patients (Fig. 5B–E). No significant differences were found in bacterial groups measured by qPCR (data not shown) between metformin‐ and nonmetformin‐treated patients.

**Table 6:** Diversity, richness and evenness indices in T2D patients and controls. Values are means and SDs

	T2D patients			Controls		
	LP	HP	*P*‐value	LP	HP	*P‐*value
*n*	11	9		16	9	
Richness	73.9 (17.9)	80.2 (22.2)	0.456	85.0 (19.9)	76.8 (19.8)	0.328
Evenness	0.70 (0.09)	0.72 (0.02)	0.787	0.70 (0.10)	0.66 (0.09)	0.354
Diversity (Shannon)	3.27 (0.48)	3.32 (0.38)	0.941	3.34 (0.56)	3.12 (0.54)	0.598
Diversity (Simpson)	0.91 (0.05)	0.91 (0.04)	0.520	0.89 (0.10)	0.87 (0.11)	0.337
Diversity (Fisher's)	24.6 (6.7)	27.1 (10.2)	0.498	30.2 (9.7)	26.3 (9.4)	0.403

There were no statistically significant differences in intestinal permeability, biochemistry variables or dietary intakes between metformin‐ and nonmetformin‐treated patients, although IL‐6 and TNF‐*α* tended to be higher (IL‐6: 12.9 ± 1.6 vs. 7.9 ± 1.3 pg/mL, *P* = 0.069; TNF‐*α*: 13.0 (5.0–22.3) vs. 5.0 (5.0–13.0) pg/mL, *P* = 0.093) and total cholesterol and LDL cholesterol tended to be lower with metformin use (total cholesterol: 3.2 ± 0.2 vs. 3.9 ± 0.3 mmol/L, *P* = 0.051; LDL cholesterol: 1.8 ± 0.1 vs. 2.4 ± 0.2 mmol/L, *P* = 0.074).

## Discussion

In this study we have measured markers of glucose tolerance, inflammation, endotoxemia, intestinal permeability, and intestinal bacterial community structure in the same individuals, investigating for the first time whether there is a potential link between intestinal permeability and glucose control/inflammation in T2D, as a first translation from the extensive and seemingly consistent animal literature. Our findings do not suggest strong links between intestinal permeability and the microbiota, inflammation and diet exist in T2D patients. Intestinal permeability did not differ significantly between NW, OW, and T2D patients, although it was numerically higher in T2D patients. This is in contrast with our previous study (Horton et al. 2014), however, a relatively large proportion of the controls were found to be above the 95% CI cut‐off for “normal” total and colonic permeability established in our previous work. Previously we established “normal permeability” in those with “normal insulin sensitivity” established by HOMA (mean HOMA IR of 1.0). However, in this study, the nondiabetic group was recruited to be a phenotypic match to the diabetes group and so had with a much broader range of background insulin sensitivity, more representative of the general population. Fat intake was significantly higher in the OW group compared with the T2D patients and a high‐fat diet has been demonstrated to induce gut barrier dysfunction (Cani et al. 2008; Stenman et al. 2012). The lower sugar and fat intakes in T2D patients suggest they may have modified their diet since being diagnosed with diabetes, however, very few significant correlations between dietary intakes and microbial abundances were found in the T2D group (data not shown).

Comparison of the LP and HP groups revealed that high permeability was associated with lower *Selenomonadales* levels and *Enterobacteriales* enrichment of the intestinal microbiota, higher fasting glucose (as a potential indicator of impaired hepatic glucose output) and elevated serum NEFA (as a proxy for adipose tissue dysfunction) in T2D. However, in contrast to the animal literature and our recent pilot study (Horton et al. 2014), there was no association between intestinal permeability and inflammatory markers. The patients in this study have a better glucose control than those in the pilot study based on fasting glucose, HbA1c and HOMA‐IR. This could potentially have made it more difficult to detect associations between outcomes. The use of medications may also have confounded the association between inflammatory markers and intestinal permeability and partly explain the lack of a significant difference in intestinal permeability between T2D patients and controls in this study. A higher proportion of T2D patients (>80% vs. 65%) was taking antihyperglycemic medication than in the pilot study by Horton et al. (2014). Also, more than 50% of the participants were taking of lipid lowering and/or blood pressure lowering medications in this study. Statins and hypotensive medications have been demonstrated to have anti‐inflammatory effects (Andrzejczak et al. 2007; Wang et al. 2011).

Although BMI and body fat percentage were not significantly different between HP and LP groups, the slightly higher BMI and body fat percentage in the T2D HP group may contributed to the elevated fasting glucose and NEFA, independently from the increased intestinal permeability. With no significant difference in HbA1c, inflammatory markers or insulin sensitivity between HP and LP groups, the clinical significance of impaired intestinal barrier function remains uncertain. However, in the healthy matched controls, increased intestinal permeability was again associated with elevated *Enterobacteriales* levels.

We observed a common feature, higher abundance of the gram‐negative bacteria *Enterobacteriales* in both HP T2D and control subgroups, suggesting *Enterobacteriales* enrichment may be mechanistically linked to increased colonic permeability irrespective of glucose tolerance status. Although gram‐negative bacteria have been suggested to influence gut barrier function by elevating LPS levels in the gut lumen (Cani et al. 2007), recent studies suggests some gram‐negative bacteria (e.g. *Akkermansia muciniphila*) may beneficial to the host (Dao et al. 2016; Plovier et al. 2016). Nonetheless, Proteobacteria and *E*. *coli* enrichment has also been observed in patients with inflammatory bowel disease which is associated with increased intestinal permeability. The role of Proteobacteria and *E*. *coli* in disease pathogenesis remains uncertain (Jensen et al. 2015; Matsuoka and Kanai 2015; König et al. 2016). However, *Enterobacteriaceae* enrichment has been observed in other clinical scenarios, such as following gastric bypass surgery (RYGB), with no detrimental effects to the host. In the case of RYGB it even concurs with resolution of T2D (Graessler et al. 2013).

T2D patients with high colonic permeability had lower abundances of *Selenomonadales* and *Prevotella*. The lower abundance of *Prevotella* may be related to the lower carbohydrate intake in the HP group. *Prevotella* is generally thought to be beneficial to the host (Wu et al. 2011), although the *Prevotella* species, *P. copri,* has been linked to insulin resistance and glucose intolerance (Pedersen et al. 2016). The role of *Selenomonadales* in human health is unclear, but it contains short‐chain fatty acid (SCFA) producing bacteria which may be beneficial in terms of gut health (Lecomte et al. 2015).

The overall higher abundance of *Enterobacteriales*, unclassified *Enterobacteriaceae* and *E*. *coli* in T2D patients compared to matched controls, may partly be due to the use of oral antidiabetic medication (Forslund et al. 2015). We confirmed this in a retrospective subgroup analysis comparing metformin with nonmetformin‐treated patients showing that these bacterial taxa were indeed enriched in metformin‐treated patients confirming findings from clinical trials (Napolitano et al. 2014; Wu et al. 2017) and other cross‐sectional studies (Karlsson et al. 2013; Forslund et al. 2015). Thus, enrichment of Enterobacteria in the T2D group compared with healthy controls may be attributed to metformin treatment and may not be a characteristic of the intestinal microbiome of T2D patients *per se*. This emphasizes the importance of taking medications into account when comparing populations with a health condition, with healthy controls. However, this remains an issue with translating into human T2D due to the almost ubiquitous use of antidiabetic medication in patients. Importantly, it must be noted that in this study the control group showed the same relationship between *Enterobacteriales* and intestinal permeability, without any confounding effects of medication. Also, a similar proportion of the T2D patients used metformin in the LP and HP groups. This indicates that factors other than metformin use may have resulted in the further increase in *Enterobacteriales* abundance in the HP T2D group.

A higher abundance of *Blautia* in the control HP group was surprising. The exact composition of this taxa may be crucial as individuals with normal glucose tolerance and T2D patients have been reported to display enrichment with different members of *Blautia* (Zhang et al. 2013). Interestingly metformin use reduced the abundance of a number of bacteria, including *Blautia*, in high‐fat diet fed mice and this was associated with improved glucose control (Shin et al. 2014).

No difference in species richness between control and diabetes patients and between HP and LP groups (Table 6) were found in this study which is in contrast to previous findings (Larsen et al. 2010; Forslund et al. 2015). Again, metformin may also have played a role by modifying the intestinal microbiota in a favorable manner. In the study by Forslund et al. (Forslund et al. 2015) in those patients taking metformin the gene richness median and distribution resembled those of the nondiabetic controls when compared to the nonmetformin‐treated diabetes patients. However, the small sample size of this study is a limitation and may explain the lack of significant differences between groups.

Further limitations to this study are primarily the hypothesis generating nature of the work and the fact only men were included in the study. However, in contrast to animal models of T2D, medication use is ubiquitous in patients with T2D, especially in the UK with respect to the prescription of statins and metformin. Understanding the implication of oral medication and the interaction between different drugs, the interactions with diet and the disease process on the microbiome is likely to be important when conducting future large scale clinical trials.

In conclusion, impaired colonic permeability was associated with a modest elevation in fasting glucose and NEFA in T2D patients. Both in nondiabetic controls and T2D, high permeability was characterized by a higher abundance of *Enterobacteriales,* however, there was no indication from the measurements conducted in this study that this increased the risk of metabolic disease in the control group. Metformin is a clear confounding factor when comparing T2D patients with healthy controls; however, despite the increase in Enterobacteria with metformin treatment, this did not have a detrimental effect on the metabolic phenotype.

## Conflict of Interest

The authors have nothing to disclose.
